# Memristor networks for real-time neural activity analysis

**DOI:** 10.1038/s41467-020-16261-1

**Published:** 2020-05-15

**Authors:** Xiaojian Zhu, Qiwen Wang, Wei D. Lu

**Affiliations:** Department of Electrical Engineering and Computer Science, The University of Michigan, Ann Arbor, MI 48109 USA

**Keywords:** Electrical and electronic engineering, Nanoscale devices

## Abstract

The ability to efficiently analyze the activities of biological neural networks can significantly promote our understanding of neural communications and functionalities. However, conventional neural signal analysis approaches need to transmit and store large amounts of raw recording data, followed by extensive processing offline, posing significant challenges to the hardware and preventing real-time analysis and feedback. Here, we demonstrate a memristor-based reservoir computing (RC) system that can potentially analyze neural signals in real-time. We show that the perovskite halide-based memristor can be directly driven by emulated neural spikes, where the memristor state reflects temporal features in the neural spike train. The RC system is successfully used to recognize neural firing patterns, monitor the transition of the firing patterns, and identify neural synchronization states among different neurons. Advanced neuroelectronic systems with such memristor networks can enable efficient neural signal analysis with high spatiotemporal precision, and possibly closed-loop feedback control.

## Abstract (web summary)

Designing energy efficient artificial neural networks for real-time analysis remains a challenge. Here, the authors report the development of a perovskite halide (CsPbI3) memristor-based Reservoir Computing system for real-time recognition of neural firing patterns and neural synchronization states.

## Introduction

In nervous systems, the collective neuron activities and firing patterns control the function, consciousness, and memory formation^1^. Revealing the features encoded in neural spike trains from the neural network will significantly advance our understanding of the working mechanism of the nervous system^2,3^. Neural probe technologies, such as patch clamp^4^, nanowire-based field effect transistor^5^, microelectromechanical (MEMS) probes^6^, and complementary metal–oxide–semiconductor (CMOS) nanoelectrode arrays^7^, are often used to record intracellular and intercellular electrophysiological activities, i.e. neural spikes, from biological neurons. Data recorded over time and over different recording sites are then transmitted and stored, and later processed in an external data-processing system that may include conventional computers and more recently artificial neural networks (ANNs)^8,9^ for analysis. Transmitting, digitizing, and storing the vast amounts of data pose severe power and throughput constraints on the neural probe design^10–12^, while the offline processing is a time-consuming process that do not allow real-time analysis. The ability to directly process neural signals at the recording sites, without having to go through pre-processing and storage, will significantly expand the capabilities of the neural probes and enhance our understanding of nervous systems, with the potential for real-time neural activity analysis and feedback in a closed loop.

Reservoir computing (RC) is a concept originally developed from recurrent neural networks (RNNs)^13^ and has recently been successfully used to implement a broad range of tasks, such as image pattern recognition, time series forecasting, and pattern generation^14,15^. Briefly, a reservoir is a dynamic system that can perform nonlinear transformations of the input signals, and project them to a high-dimensional space (represented as the reservoir states). The nonlinear transformation allows the original features, often in time domain, to be mapped as features in the reservoir states which can then be further processed by a small, trained linear neural network (termed the readout layer)^14^.

A key feature of the reservoir is the fading memory^14^, i.e. short-term memory property, which states the reservoir state depends not only on the present inputs but also on inputs from the recent past (but not the far past). The fading memory effect is key for an RC system to extract and analyze temporal features in the input data. In particular, since information in neural spike trains is mainly encoded in the temporal domain, we expect neural signals to be well suited for RC hardware systems. More specifically, dynamic memristors with inherent short-term memory effects have recently been successfully utilized as reservoirs for temporal data processing^15^. Beyond benefits such as having a simple device structure that allows easy fabrication and integration, the characteristics of a memristor device can be tailored by carefully engineering the switching material and optimizing the device structure^16–19^, allowing one to develop memristors with desired operation voltages and dynamics for different applications. To this end, memristor-based RC systems offer intriguing opportunities to be integrated with the neural probe for on-site, real-time neural signal processing.

In this work, we experimentally demonstrate the possibility of neural data analysis using a memristor-based RC system. A perovskite halide-based memristor with very low switching voltage (<100 mV) and current (1–100 nA) and an inherent short-term memory effect is developed and used as the reservoir. Using emulated neural spike signals, we show that an RC system based on such devices can potentially directly process neural spikes, and is capable of implementing important tasks, such as real-time recognition of neural firing patterns and neural synchronization states.

## Results

### Low voltage dynamic memristor

**Fig. 1:**
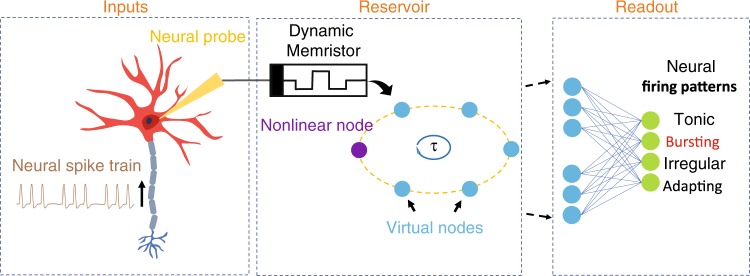
Schematic showing the concept of a memristor-based RC system for neural activity analysis. Inputs: Neural spikes collected from a firing neuron are directly used as inputs to excite the memristor. Reservoir: The reservoir space is further expanded by the concept of virtual nodes to help process complex temporal inputs. Readout: A simple ANN is used as readout layer of the reservoir to produce the final output (e.g. recognizing “Bursting” firing pattern in this example).

In the proposed RC system for neural activity analysis (Fig. 1), the memristor device serving as the reservoir plays a key role. A memristor is essentially a two-terminal resistive device whose conductance can be modulated by an electrical input, normally through the redistribution of ions^16–19^. For example, under an electric field during the SET process, the migration of oxygen ions in oxide films can generate oxygen vacancies (V_O_s) acting as n-type dopants to form local conduction channels and increase the device conductance. After removing the electric field, the V_O_s in devices with low activation energies and weak conduction channels can diffuse and result in the spontaneous rupture of the conduction channels, leading to the short-term memory effects^20,21^. RC systems based on such dynamic memristors have been successfully demonstrated recently^15^.

Notably, most existing memristors, e.g. oxide-based^22^, require a high programming voltage (e.g. ~1 V or higher) and a high programming current (e.g. >10 μA), due to the relatively high activation energy barrier for V_O_ migration and the high conductivity in the conduction channels, respectively. In contrast, neural spikes are electrical pulses with very low-power (<100 fJ) and voltage amplitude (~100 mV)^1^. To allow memristors to directly respond to neural spikes without pre-processing, devices with much lower programming voltage and current are needed. Recent experimental and theoretical studies suggest that low activation energy for halogen ion migration can be obtained in perovskite halides (e.g. 0.17 eV for iodine ion in CH_3_NH_3_PbI_3_ (ref. ^23^) and 0.25 eV for bromide ion in CsPbBr_3_ (ref. ^24^)) that possess low intrinsic defect concentrations (e.g. 10^16^–10^17^ cm^−3^ for polycrystalline CH_3_NH_3_PbI_3_ (ref. ^25^) and ~10^15^ cm^−3^ for polycrystalline CsPbI_3_ (ref. ^26^)), indicating memristors based on such films may exhibit low operation voltage and current needed for direct neural data processing.

**Fig. 2:**
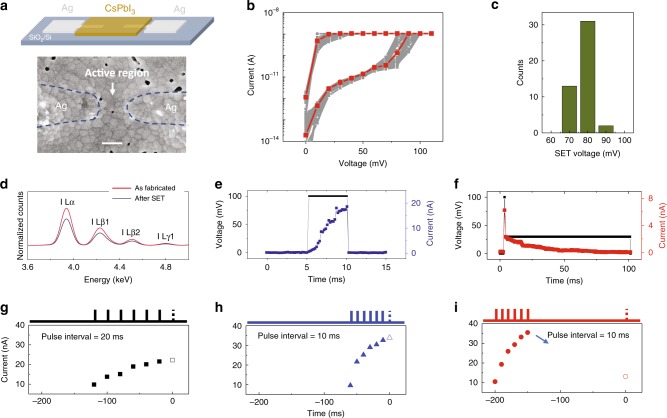
CsPbI_3_-based memristor with low programming voltage and short-term memory effect. **a** Schematic (upper) and SEM image (bottom) of an as-fabricated Ag/CsPbI_3_/Ag memristor device. Scale bar: 200 nm. **b** Current–voltage (*I–V*) characteristics of the device during 46 SET processes. A typical *I–V* curve is highlighted in red. **c** Histogram of the SET voltage extracted from **b**. **d** EDX spectra of a device showing characteristic Iodine X-ray peaks (L series) before (red curve) and after (blue curve) the SET process. The intensity of the Iodine X-ray peaks is reduced after SET process. **e** Evolution of the device current (blue curve) with time during the application of a stimulation pulse (black curve, 100 mV, 5 ms, started at *t* = 5 ms). **f** Evolution of the device current (red curve) with time during the application of a stimulation pulse (100 mV, 2 ms, started at *t* = 3 ms) followed by a long read pulse (30 mV, 100 ms, black curve). **g**–**i** Current responses in a Ag/CsPbI_3_/Ag device subjected to seven identical stimulation pulses (100 mV, 2 ms) with different pulse interval conditions. The device currents recorded at *t* = 0 ms are represented as open symbols.

In this study, we chose CsPbI_3_ as the switching material. CsPbI_3_ is a member of perovskite halide family, and offers good chemical stability in ambient in contrast to other perovskite halides^26,27^. Figure 2a shows the schematic and SEM image of an as-fabricated planar Ag/CsPbI_3_/Ag memristor device (see “Methods” section). Memristive effects were examined by applying DC sweep voltages to study the conductance evolutions. After forming and SET processes (Supplementary Fig. 1), low voltage and volatile memristive effects were observed. Figure 2b shows the current–voltage (*I–V*) curves of a device during voltage sweeps (0 mV → 100 mV → 0 mV), where the device initially at HRS (~1 × 10^10^ Ω, read at 30 mV) was switched to the LRS (~1 × 10^7^ Ω) at a low SET voltage (e.g. 80 mV for the red curve) and a compliance current of 1 nA. The LRS was volatile and returned to the HRS after the sweep voltage was removed. Statistics results extracted from the *I–V* curves in Fig. 2b show that the average SET voltage is ~80 mV (Fig. 2c). Energy-dispersive spectroscopy (EDS) measurements (Fig. 2d) revealed that the iodine concentration in the CsPbI_3_ film was decreased after repeated SET processes, suggesting the memristive effect likely originates from the generation of V_I_s under the applied electric field. This result is consistent with the findings obtained in other perovskite-based memristors, including Ag/CH_3_NH_3_PbI_3_/Ag devices that exhibited increase/decrease in V_I_ concentration that correspond to the increase/decrease in the device conductivity^23^, and Ag/CsPbBr_3_/Ag devices that showed increased V_Br_ concentration after SET processes^28^. Moreover, redox reaction peaks observed during cyclic-voltammetry measurements suggest possible electrochemical reactions between the silver electrodes and the iodine ions removed from the CsPbI_3_ film^23,29^ (Supplementary Fig. 2), further supporting the hypothesis that the formation of V_I_s leads to the memristive effect.

Response of the memristors to low-voltage electrical pulses was then analyzed. Evolution of the device current with a single voltage pulse (100 mV, 5 ms) is shown in Fig. 2e. The device current was found to nonlinearly and continuously increase from <1 nA to ~20 nA during the pulse stimulation, indicating successful programming with pulses at 100 mV. Even lower programming current (~1 nA) can be obtained by reducing the programming pulse width (100 mV, 500 μs) (Supplementary Fig. 3). The energy consumption was estimated to be ~50 fJ, comparable to that of an actual neural spike^1^. The short-memory behavior of the LRS was also examined by pulse tests. After being excited by a stimulation pulse (100 mV, 2 ms), a gradual decay of the read current (read at 30 mV) was detected with a typical relaxation time of 100 ms (Fig. 2f and Supplementary Fig. 4). We note that actual neural signals are more than simple spikes^1^. To study the memristor response to such a complex waveform, we carefully emulated the action potential with a voltage waveform, as shown in Supplementary Fig. 5a, b. The waveform is applied to the Ag/CsPbI_3_/Ag memristor device, and the device conductance evolution is then recorded. The device was successfully switched to a high conductance state, followed by a gradual decay, indicating that the device can potentially be directly driven by actually recorded neural signals (Supplementary Fig. 5b). When compared to the device response to a simple digital pulse waveform, the device shows qualitatively similar responses (Supplementary Fig. 5c). These results suggest that the device response to the complex action potential recording can be reasonably emulated by simple digital pulses with proper width and amplitude (i.e. 100 mV, 2 ms). In our study, we thus use digital pulses with these parameters to emulate the effect of actual neural spikes acquired in a nervous system to ease the experimental implementation. In practical applications, we expect the memristor device will be able to directly response to the complex action potentials without the need of recording and digitization.

The ability of the Ag/CsPbI_3_/Ag memristor device to act as a reservoir was then carefully evaluated. We show that the Ag/CsPbI_3_/Ag memristor devices offer the properties of a reservoir, including internal dynamics, nonlinearity, fading memory, separability and echo state property^14^ (Supplementary Figs. 6–10 and Supplementary Note 1). For example, in a test of the echo state property, three pulse trains consisting of seven identical stimulation pulses (100 mV, 2 ms) were applied to the device with different pulse intervals, and the device state after the stimulation was measured, as shown in Fig. 2g–i. It was found that the device current at *t* = 0 ms (marked by the open symbol in each figure and measured by a 100 mV programming pulse), increases with the rate of the pulses applied between −120 and 0 ms (Fig. 2g–h), but is not affected by pulses applied earlier than −150 ms (Fig. 2i). These results are consistent with dynamic memristor behaviors that have been discussed previously, e.g. in WO_*x*_ memristors^15,20^, and suggest that the present device state reflects the temporal features in the inputs in the recent past but not in the far past, allowing the Ag/CsPbI_3_/Ag memristors to act as reservoirs in RC systems (more discussions can be found in Supplementary Note 2).

### Neural firing pattern recognition

**Fig. 3:**
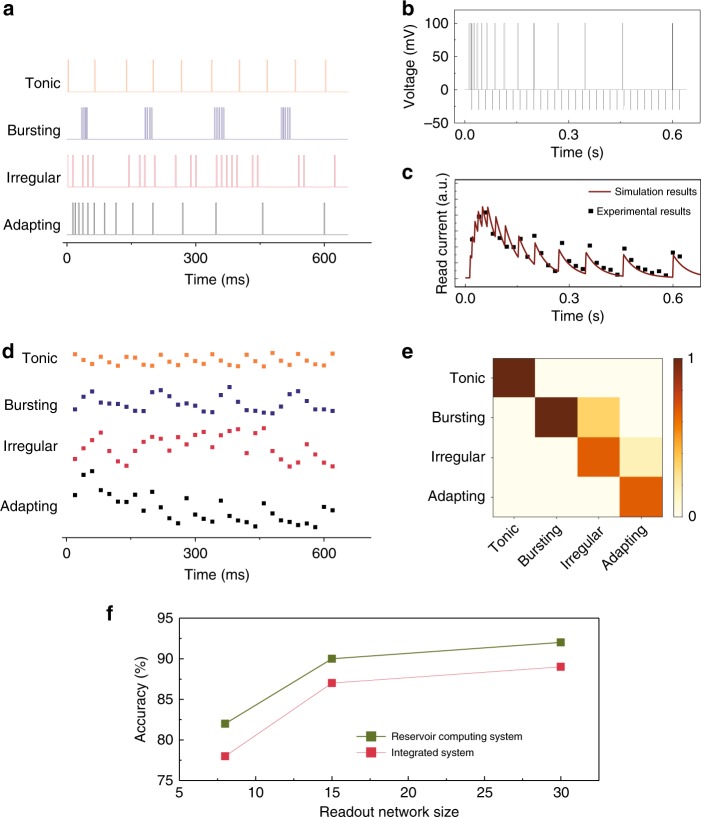
Neural firing pattern recognition. **a** Typical spike trains corresponding to four common neural firing patterns. **b** Waveform of a spike train applied to the memristor during the measurements, including spike inputs (the “Adapting” pattern in this example) and the read pulses (−30 mV, 0.5 ms, with an interval of 20 ms). **c** Evolution of the read current (black dots) measured from the device when excited by the spike train in **b**. The experimental results agree well with the simulation results (solid curve) based on a dynamic memristor model^15,20^. **d** Evolution of the memristor read current for the spike trains shown in **a**. **e** False color confusion map showing the experimentally obtained recognition results. The occurrence probability for each firing pattern is represented by the colors shown in the color scale. **f** Simulated neural firing pattern recognition accuracy at different readout layer sizes, for the integrated system that integrates the input data over a fixed time period, and the memristor-based reservoir system.

An RC system based on Ag/CsPbI_3_/Ag memristor devices and an ANN readout layer was then used to perform neural firing pattern recognition tasks. Four common neural firing patterns^30^, namely, “Tonic”, “Bursting”, “Irregular”, and “Adapting”, were chosen for the study. Typical spike trains corresponding to these patterns are schematically shown in Fig. 3a. Briefly, “Tonic” pattern corresponds to low-frequency spikes with a constant interval; “Bursting” pattern corresponds to multiple groups of high-frequency spikes with a constant inter-group interval; “Adapting” pattern corresponds to spikes with gradually increased intervals; “Irregular” pattern corresponds to spikes that fire irregularly. To facilitate the tests, we manually generated spike trains composed of square-wave voltage pulses (100 mV, 2 ms) that emulate the four patterns (see “Methods” section). These spike trains were used to excite a Ag/CsPbI_3_/Ag memristor, whose conductance state was measured by a read pulse (−30 mV, 0.5 ms) every 20 ms. The circuit diagram for the measurement is shown in Supplementary Fig. 11. As an example, Fig. 3b shows the waveform of the pulses corresponding to the “Adapting” pattern combined with the read pulses. The corresponding experimentally obtained read current values (black dots) are shown in Fig. 3c. One can see that the read current quickly increases at the beginning when the spike frequency is high, then decreases as the spike stimulation becomes less frequent, correlating well with the patterns in the spike train. The experimental results also agree well with simulations (solid line) based on a dynamic memristor model^15,20^ (Supplementary Note 3), allowing the device behaviors to be accurately predicted. Distinctive responses to the four different types of spike trains were clearly observed, as shown in Fig. 3d, allowing the memristors to be used as reservoirs for analyzing the spike trains.

We then tested whether the memristor-based RC system can be used to classify the four different spike inputs. To increase the reservoir size based on a single physical device, we employed the concept of virtual node^14^. Briefly, during the application of delayed input signals to the physical node, the physical node is dynamically excited, and these excited states recorded at pre-determined time steps create a chain of virtual nodes in time domain and reflect the temporal feature in the inputs. Specifically, following ref. ^31^, during the application of a long pulse stream to a single memristor device, instead of only recording the final conductance state after finishing feeding the complete pulse stream, we sampled and recorded the conductance state of the memristor device after each defined time interval, i.e. 20 ms. Therefore, for a pulse stream with a length of 620 ms, we can produce 31 virtual node states in total, which were measured from the same memristor device (the physical nonlinear node) at selected time steps, i.e*. t* = 20 × *n* (ms), *n* = 1–31. Each virtual node state is influenced by the previous node states along with the inputs in the recent past and at the present stage. These 31 recorded virtual node states from a single memristor device collectively form the reservoir states and are fed to a simple fully connected (FC) neural network (31 × 4) acting as the readout layer. Since the temporal features in the input spike trains are converted into features in reservoir states, pattern recognition can be achieved by only training the readout layer. Afterwards, the ability of the RC system to analyze the neural firing patterns was tested both through simulation (Supplementary Figs. 12 and 13) and in experiment (Supplementary Fig. 14). Figure 3e shows the experimentally obtained classification results plotted in a confusion map, corresponding to an overall recognition accuracy of ~87.0%. Additional control studies show that, at the same readout network sizes, the RC system systematically outperforms alternative approaches of using analog or digital circuits to produce an integrated signal of the input over a pre-determined time period (Fig. 3f), since the integrated approach cannot distinguish the temporal sequence of spikes during the integration period (Supplementary Note 4).

**Fig. 4:**
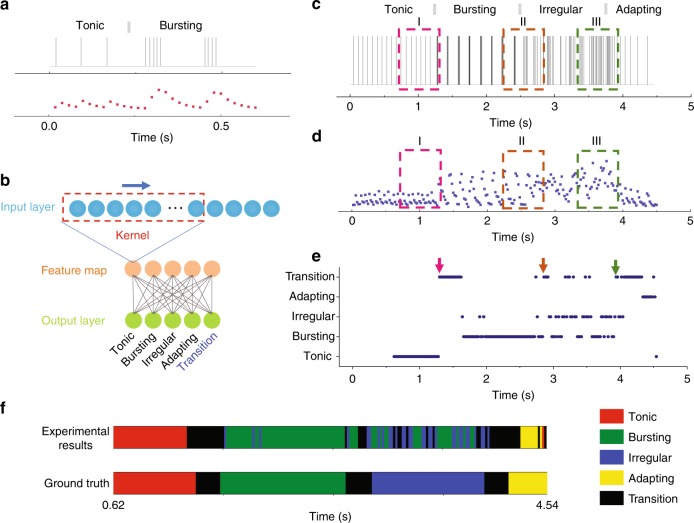
Real-time analysis of neural firing pattern evolution. **a** A spike train showing “Tonic → Bursting” transition (upper), and the corresponding simulated device current (bottom). **b** The bilayer CNN used as the readout layer. A 27 × 1 kernel is used to project the reservoir states onto a 5 × 1 feature map, which is then applied to the fully connected layer for firing pattern recognition. **c** and **d** A streaming spike train used for evaluation **c**, and the corresponding experimentally measured device responses **d**. The gray bars mark the transition between different patterns. **e** Output from the RC system for the input stream in **a**. The arrows indicate the moments when the pattern transitions began to be detected. **f** Experimental results from the RC system (upper) vs. ground truth (bottom), represented by color maps.

Beyond pattern recognition, real-time detection of firing pattern changes in a streaming spike train is highly desirable^32^, as it facilitates studies such as how neural activities respond to a stimulus. Figure 4a shows one such example of “Tonic → Bursting” transition. Below, we show the memristor-based RC system can perform real-time pattern recognition as well as detecting firing pattern changes from streaming spiking inputs. A bilayer convolutional neural network (CNN) was used as the readout layer in this case, as shown in Fig. 4b. Specifically, the first layer of the readout CNN uses a 27 × 1 convolution kernel with a stride of one to project an input of 31 reservoir state values (corresponding to 620 ms in history) onto a 5 × 1 feature map, which is then applied to the second layer, a 5 × 5 perceptron, for classification. The convolution layer allows the system to analyze the firing pattern within a “patch” of 27 reservoir states (540 ms), as well as evolution between the patches up to another 100 ms that is within the relaxation time (~100 ms) of the memristor, i.e. from −620 to −540 ms. The five output neurons correspond to the four firing patterns and a “transition” neuron that identifies the transition between any two of the four firing patterns.

We first trained the RC system using the simulated device responses, and evaluated the real-time network performance with streaming neural firing patterns. An example of experimentally measured memristor response to a streaming spike train is shown in Fig. 4c and d, with clear correlations to the firing patterns and transitions. Figure 4e shows the outputs from the network over time. The arrows highlight the moments when the pattern transitions were detected by the network, corresponding to the transitions highlighted by the dashed boxes in Fig. 4c, d, such as I “Tonic → Bursting”, II “Bursting → Irregular” and III “Irregular → Adapting”. The prediction results for the entire spike train and the manually labeled ground truth are shown in Fig. 4f. One can see that most of the prediction results are consistent with the expert-labeled ground truth results, with the exceptions that the “Irregular” patterns were often misclassified as “Bursting” or “Transition” patterns. Indeed, it can be argued that spikes in the “Irregular” pattern is composed of various high-frequency and low-frequency patterns, which can be reasonably identified as “Bursting” or “Transition” patterns, and the labeling of “Irregular” in these cases is a result of human bias. Nevertheless, these results clearly demonstrated that the memristor-based RC system can efficiently resolve the neural firing pattern transitions in the majority of cases and can be used to monitor neural activity evolutions in a real-time fashion. One such example of real-time firing pattern recognition can be found in Supplementary Movie 1.

### Real-time analysis of neural synchronization states

**Fig. 5:**
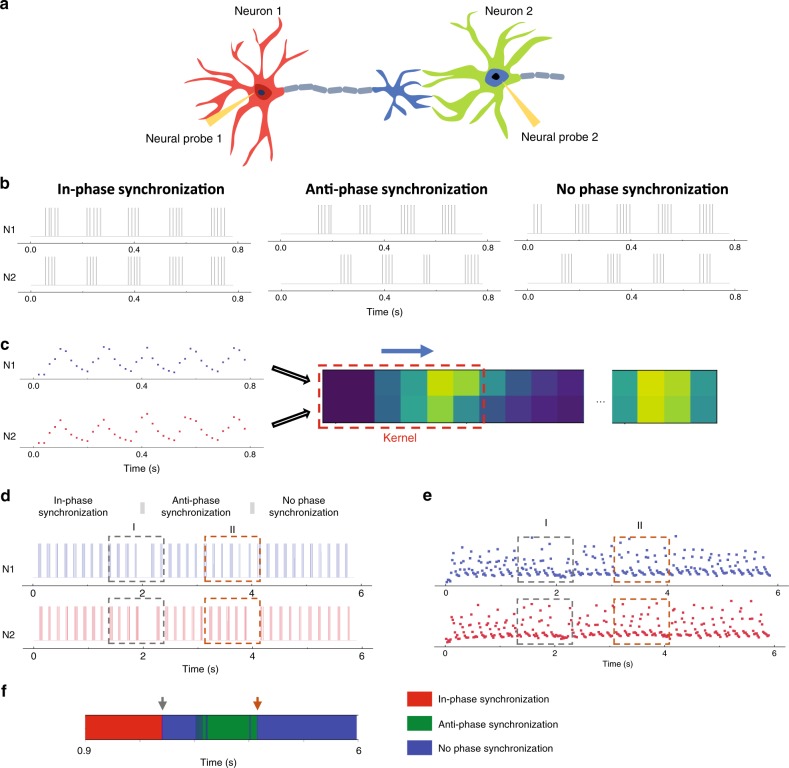
Real-time analysis of neural synchronization. **a** Schematic showing two interacting neurons, each recorded by a neural probe. **b** Typical spike trains that form in-phase synchronization (left), anti-phase synchronization (middle), and no phase synchronization (right) for two neurons N1 and N2. **c** Schematic of the RC setup. The reservoir states from two memristors form a two-row matrix that are applied to the readout layer. A 6 × 2 kernel convolutes the inputs with a stride of two to produce a 20 × 1 feature map, which is then processed by the 20 × 3 perceptron (not shown) in the readout layer to produce the final output. **d** and **e** Spike trains showing synchronization states evolving from in-phase synchronization, anti-phase synchronization to no phase synchronization **d**, and experimentally measured device states in two memristors **e**. The gray bars in **d** mark the transition between different synchronization states. **f** Classification results from the RC system. The arrows indicate the moments when the synchronization state transitions are detected.

The ability to detect neuron firing patterns directly, in real-time, allows us to further test the ability of the memristor-based RC system to analyze multi-neuron firing activities. Specifically, synchronized neural firing has been widely observed in biological neural networks and plays an essential role in facilitating neural communications, synaptic plasticity, and memory formation^33,34^. Here we aim to use the memristor-based RC system to analyze the synchronization states between two neurons (Fig. 5a).

For example, Fig. 5b shows spike trains from two neurons (N1 and N2) firing “Bursting” patterns that correspond to in-phase synchronization (left), anti-phase synchronization (middle) and no phase synchronization (right)^35^. In-phase and anti-phase synchronization refer to the simultaneous and alternative firing in two neurons, which can be caused by the formation of excitatory and inhibitory synaptic connections between the neurons, respectively^33^. In contrast, no phase synchronization refers to spike firings without correlations. We emulated various spike trains corresponding to the three types of neural synchronization states and trained the RC network using simulated device responses. Specifically, we utilized two memristors as the reservoir (with one receiving a spike train from a specific neuron). The reservoir states then formed a two-row matrix, e.g. 45 × 2 for a 900 ms time window, and were applied to the readout layer (Fig. 5c). The readout layer was based on a CNN with a 6 × 2 convolution kernel and a stride of two in the first layer. The resulting 20 × 1 feature map was then fed to the second layer, a 20 × 3 perceptron, for analysis. After training the readout network, the performance of the RC system was investigated experimentally. For example, in one test we generated spike trains (Fig. 5d) evolving from in-phase synchronization, anti-phase synchronization to no phase synchronization. The corresponding reservoir states from the two memristor devices are shown in Fig. 5e, and the final RC network output is shown in Fig. 5f. The system can successfully recognize the neural synchronization state at different moments, and also detect the transitions between different synchronization states (represented by no phase synchronization), demonstrating the capability of the memristor-based RC system to resolve correlations between spikes from different neurons. Further simulation results suggest that the system can be expanded and used to analyze the synchronization states among multiple neurons (e.g. four neurons shown in Supplementary Fig. 15), providing a promising means to study the neuron activity correlations in large biological neural networks.

## Discussion

In this work, we aim to show that memristor-based reservoirs with tailored operation conditions can be obtained by carefully choosing the switching material and designing the device structure. These systems and devices in turn create new opportunities for critical applications, such as neuroscience and engineering. Specifically, we demonstrated that by choosing proper ion species, ion migration energy barrier, and the intrinsic defect density, one can develop dynamic memristors, e.g. Ag/CsPbI_3_/Ag, with ultralow switching voltage (<100 mV) and switching current (~nA), where both the low programming voltage and low programming current are necessary if such devices need to be directly interfaced with biological neural signals. We further show that with these unique properties, these devices can be potentially directly driven by actual biological neural spikes and interfaced with neural probes for neural firing pattern recognition and neural synchronization analysis.

Emulated neural spiking patterns were used for training and analysis in this study. Considering that actual neural spike patterns may contain diverse and complex temporal features at different timescales, dynamic memristors with different relaxation rates are desired and need to be carefully designed. Improvements in the readout layer, as well as training algorithms are excepted to further enhance the RC system performance. By integrating the low-voltage RC system with recently developed intracellular neural probes, for instance, large-scale nanoelectrode arrays that can simultaneously perform intracellular recordings from thousands of connected mammalian neurons^7,36^, real-time analysis of the interactions among many neurons in a large biological neural network may become feasible. Such large-scale implementations, however, would still require the development of new RC computing algorithms and the optimization of the memristor hardware.

Beyond electrophysiological data recording and processing, the RC artificial network, which was originally inspired by neurobiology and offers functionalities resembling that of biological systems^37^, can possibly play a more active role though the interactions with the biological neural network^38^. For example, with the ability to be directly excited by neural spikes, these electronic networks may be considered as an extension of the biological neural network, and offer additional resources for tasks such as recognition and memory formation.

## Methods

### Device fabrication

Ag (200 nm)/Au (100 nm)/Ti (5 nm) electrodes with ~200 nm spacing were fabricated on a SiO_2_/Si substrate by photolithography and e-beam evaporation, followed by a lift-off process. CsPbI_3_ precursor solution with 40 wt% concentration was prepared by mixing the weighted CsPbI_3_ powder (99.5%, Xi’an Polymer Light Technology Co., Ltd.) in N,N-dimethylformamide (DMF, 99.8%, Sigma-Aldrich) and stirred at 70 °C for 2 h. The Ag electrodes were treated by CF_4_ plasma for 30 s before spin-coating the CsPbI_3_ film. The precursor solution was first spin-coated on the substrate at 4000 rpm for 60 s, during which ~100 μL of toluene (99.8%, Sigma-Aldrich) was applied at ~11 s. The samples were then annealed on a hot plate at 100 °C for 30 min.

### Measurements

Electrical characterizations were performed using a probe station and a Keithley 4200s semiconductor parameter analyzer system with source measure units (SMU) and pulse measure units (PMU). SEM images and EDX spectra of the devices were acquired from a Hitachi SU8000 system at 10 kV with emission current 7 µA.

### Generation of neural spike trains for pattern recognition

Neural spikes are emulated by square wave electrical pulses (100 mV, 2 ms). The duration of each spike train is 620 ms. The design rules of the spike trains are described below. For “Tonic” patterns, each spike train is composed of low-frequency pulses with a constant interval. The average inter-pulse interval is between 60 and 80 ms for different samples. For each sample, the inter-pulse interval follows a Gaussian distribution, having a standard deviation of 5%. For “Bursting” patterns, each spike train is composed of multiple groups of high-frequency pulses, containing 3–6 pulses in each group. The inter-pulse interval in each group is ~10 ms, and the inter-group interval is between 100 and 200 ms for different samples. Both inter-pulse interval and inter-group interval follow the Gaussian distribution, having a standard deviation of 10% and 8%, respectively. For “Irregular” patterns, each spike train was divided into 50 segments in time domain, with the duration of each segment being 12 ms. The probability of having a spike in each segment is 50%. For “Adapting” patterns, each spike train is composed of pulses with a gradually increased interval. The interval between the first and second pulse is 6 ms. For the subsequent pulses, the interval is increased by 30% (with a standard deviation of 5%) each time. For “Transition” patterns, each spike train is composed of two shorter spike trains in series that correspond to any two of the four firing patterns. The length of each shorter spike train is between 25% and 75% of the overall spike train.

### Generation of neural spike trains for synchronization state analysis

Spike trains with “Bursting” patterns were used for synchronization state study. Each sample is composed of multiple spikes trains, determined by the number of neurons in the study. For in-phase (anti-phase) synchronization state, the pulses in each pulse train occur simultaneously (alternatively) in time domain, with a standard deviation of 10%. For no phase synchronization state, the pulses in the spike trains are not correlated in time domain.

### Training and testing the readout layers

For neural firing pattern recognition (Fig. 3), the readout layer is a FC neural network (31 × 4) with four output neurons corresponding to the “Tonic”, “Bursting”, “Irregular”, and “Adapting” patterns. For training, we simulated the conductance evolution behaviors of the memristor with 1600 spike trains, corresponding to different patterns (400 examples for each pattern). 80% of the simulation results were chosen for training. A supervised learning algorithm based on logistic regression was used to train the readout layer. Specifically, the reservoir states were fed to the readout layer in the form of a 31 × 1 input vector. The input vectors were multiplied with the weight matrix stored in the FC neural network, followed by applying a sigmoid activation function to calculate the probability values for the four output neurons. A cost function was used to measure the error between the actual output values and the desired output values. By using the gradient descent training rule, the weight matrix was adjusted to minimize the cost function. After training, the remaining 20% of the emulated spike trains were used for validation, using both simulated and experimentally obtained memristor responses.

A bilayer CNN was used as the readout layer for the streaming spike analysis (Fig. 4). Two thousand emulated spike trains corresponding to five different firing patterns were used to train the readout layer, through supervised learning based on the logistic regression method, similar to training of the readout layer for pattern recognition. A bilayer CNN was used as the readout layer for neural synchronization state analysis (Fig. 5). Seven thousand two hundred groups of emulated spike trains corresponding to three different synchronization states were used to train the readout layer following a similar approach described above.

## Supplementary information

Supplementary Information

Description of Additional Supplementary Files

Supplementary Movie 1

## Data Availability

The data that support the plots within this paper and other findings of this study are available from the corresponding author upon reasonable request. The source data underlying Figs. 2b–i, 3c, d, f, 4d, e and 5e and Supplementary Figs. 2–4, 5b, c and 7–10 are provided as a Source Data file.

## Source Data

Source Data
