# Supplementary material for: Memristor networks for real-time neural activity analysis
Source: Nat Commun. 2020 May 15;11:2439. doi: 10.1038/s41467-020-16261-1 (PMC7228921; doi:10.1038/s41467-020-16261-1)
Supplement: Supplementary file 1 — Supplementary Information [file 41467_2020_16261_MOESM1_ESM.pdf]

Supplementary Information

Memristor networks for real-time neural activity analysis

Zhu et al.

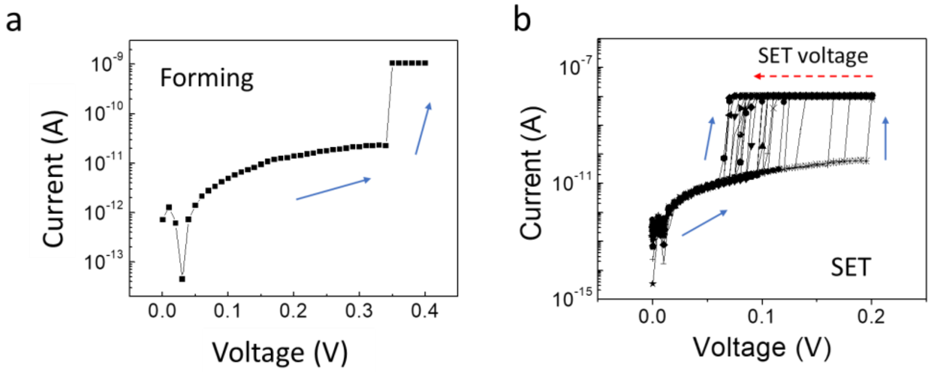

Supplementary Figure 1 Current-voltage (*I*-*V*) characteristics of a Ag/$CsPbI_3$/Ag memristor during "Forming" (**a**), and SET (**b**) processes. The blue arrows indicate the direction of the current changes. In the Forming process, the voltage was scanned from 0 to 0.4 V, where the device current jumped to the compliance current (10^{-9} A) at ~0.33 V. Similar effects can be observed during subsequent voltage sweeps (0 ⟶ 0.2 V) in SET processes, where the SET voltage gradually decreases and is eventually reduced to ~80 mV (indicated by the red dashed arrow in **b**).

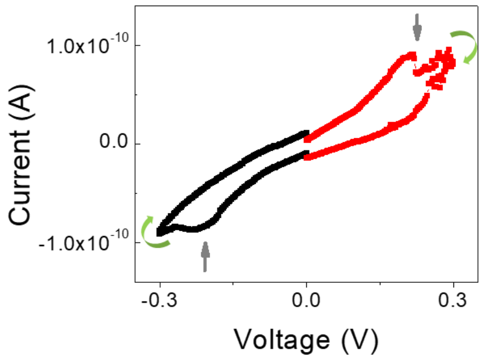

Supplementary Figure 2 *I*-*V* curve of an as-fabricated device during Cyclic-Voltammetry measurement (0 V → 0.3 V → 0 V → -0.3 V → 0 V). Note that the applied voltage was intentionally limited to not induce the Forming process. The green arrows indicate the current change direction. Redox reaction peaks were observed at around 0.22 and -0.22 V (marked by gray arrows), indicating the occurrence of electrochemical reactions in the device under the voltage sweep[1, 2].

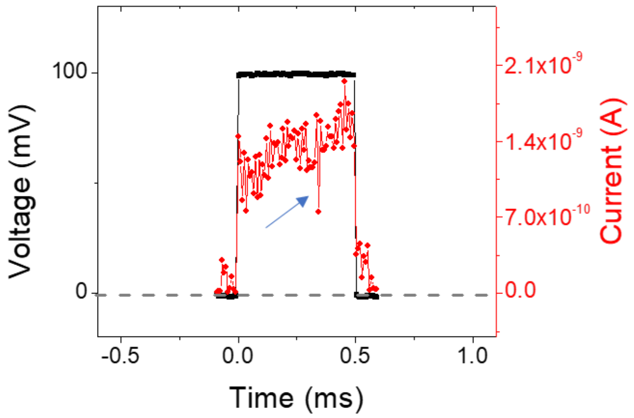

Supplementary Figure 3 Evolution of the current (red curve) with time in a device during the application of a short programming pulse (100 mV, 0.5 ms, black curve). The device underwent a SET process during programming, indicated by the gradually increased (blue arrow) device current.

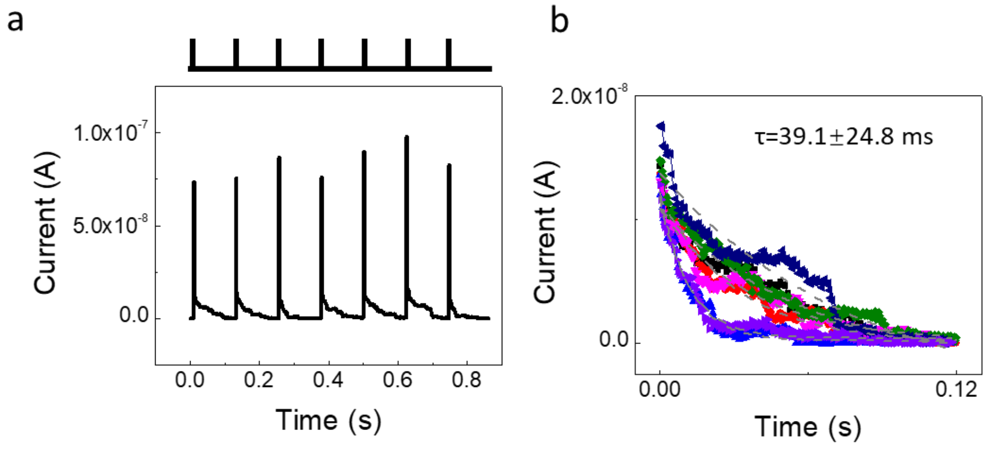

Supplementary Figure 4 Short-term memory effect in a typical Ag/$CsPbI_3$/Ag device. **a.** Current responses in the device subjected to stimulation pulses (100 mV, 2 ms) with an interval of 120 ms. A read voltage of 30 mV was applied during the pulse interval to monitor the device current. **b.** Evolution of the read current over time, measured after each programming pulse in **a**. Gradual decay of the read current was observed in each case, which can be fitted (gray dashed lines) by the stretched-exponential function ($I = I_0 \exp(-(\frac{t}{\tau})^{\beta})$), I_0, τ and β represent the prefactor, characteristic decay time constant and the stretch index, respectively[3]. The characteristic decay time constant in this measurement is 39.1±24.8 ms.

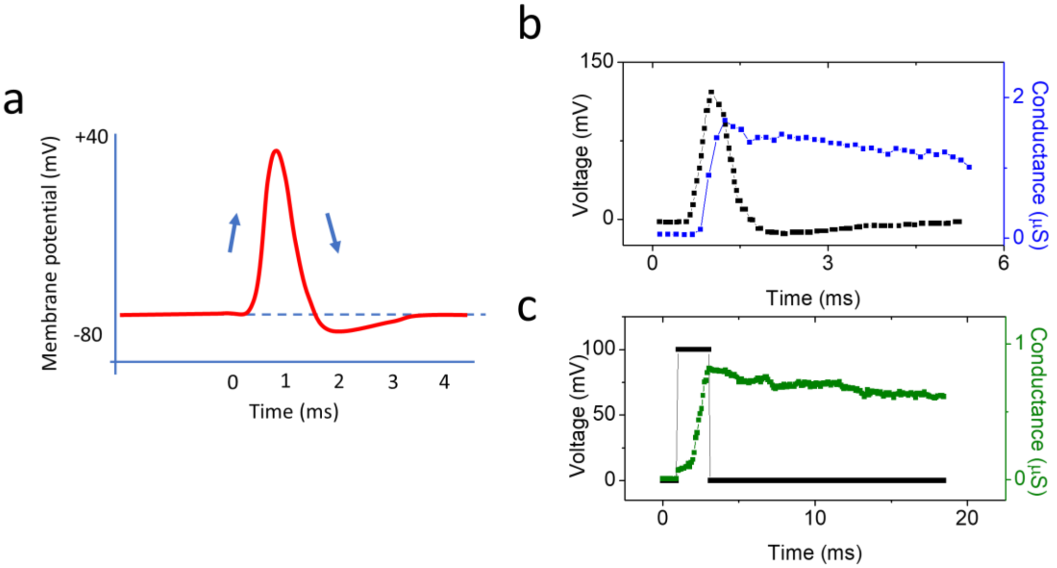

Supplementary Figure 5 a, schematic of an actual action potential. **b**, Evolution of memristor conductance (blue dots) when subjected to an electrical waveform that emulates the complex shape of the actual action potential (black dots). **c**, Evolution of memristor conductance (green dots) when subjected to a simple digital electrical waveform with properly chosen parameters (black dots). Similar device response can be obtained using the simple digital form with properly chosen parameters.

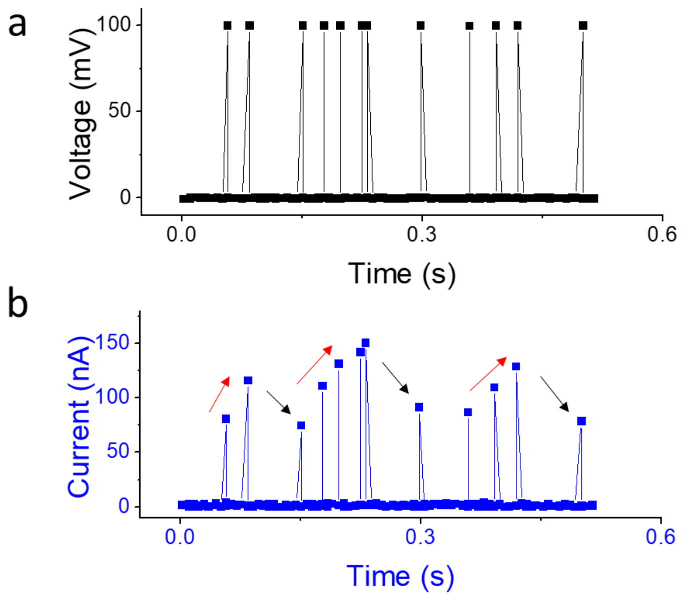

Supplementary Figure 6 a, A pulse stream composed of identical electrical pulses (100 mV, 2 ms) with different pulse intervals. **b,** Dynamic response of a Ag/$CsPbI_3$/Ag memristor to the pulse stream. The device conductance is a function of the temporal patterns of the input stream, as indicated by the red and black arrows.

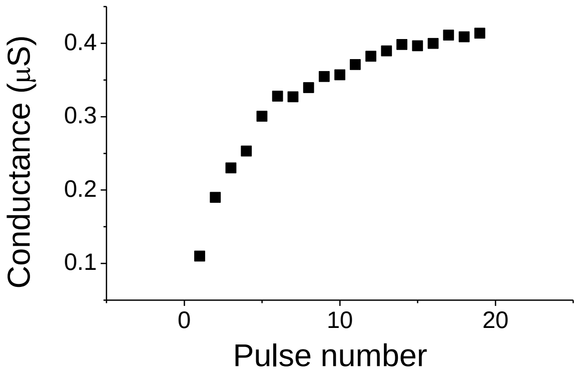

Supplementary Figure 7 Ag/$CsPbI_3$/Ag memristor conductance as a function of the number of applied electrical pulses (100 mV, 2 ms, with an interval of 20 ms), showing nonlinear conductance changes.

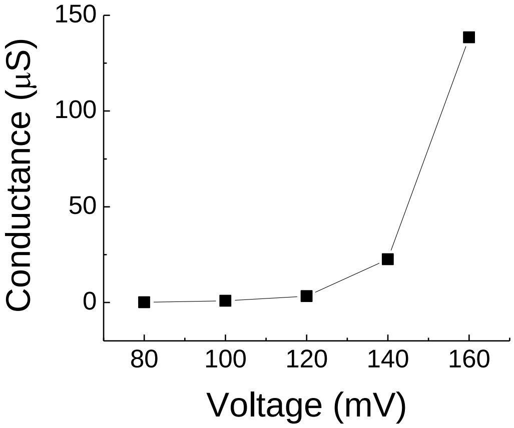

Supplementary Figure 8 Ag/$CsPbI_3$/Ag memristor conductance as a function of the amplitude of the applied electrical pulse (80-160 mV, 2 ms), showing nonlinear conductance changes.

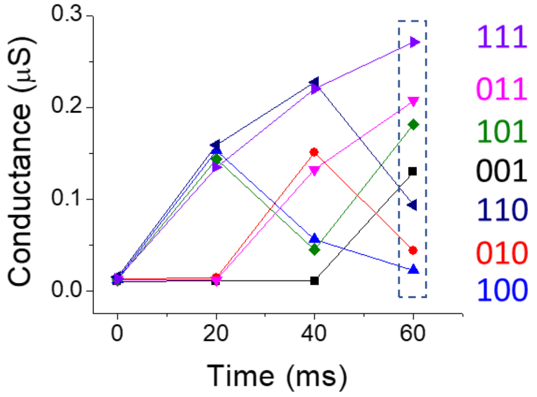

Supplementary Figure 9 Evolution of the memristor conductance during different pulse streams. The final device conductance states (marked in the dashed box) show clear separation, reflecting the temporal patterns of the pulse streams.

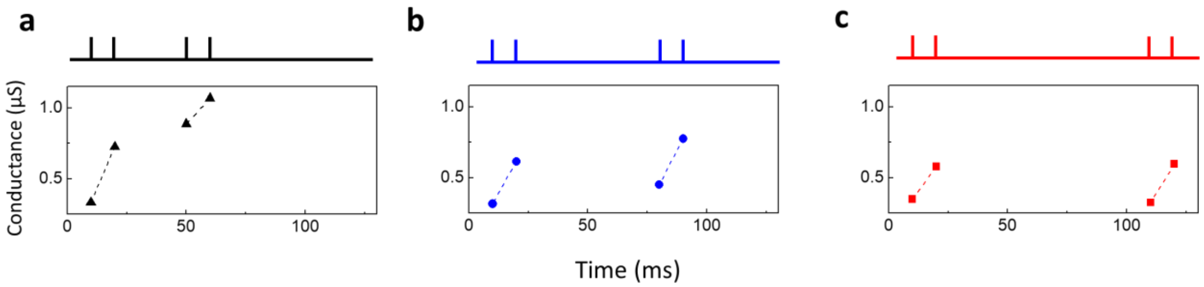

Supplementary Figure 10 Conductance of a Ag/$CsPbI_3$/Ag memristor (bottom panels) excited by two electrical pulse sequences (top panels), with increasing interval between the two sequences. Each pulse sequence consists of two identical electrical pulses (100 mV, 2 ms, 10 ms interval). The interval between the two sequences increases from 30 ms (a), to 60 ms (b) and 90 ms (c).

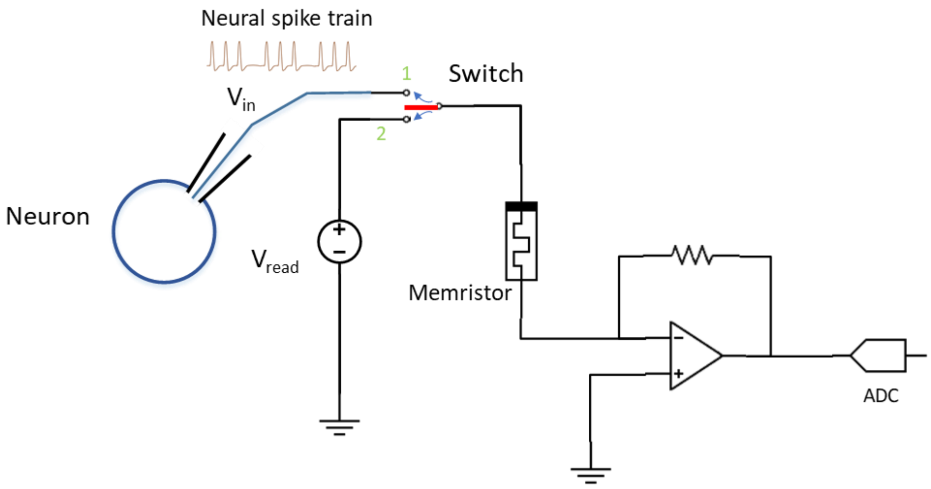

Supplementary Figure 11 Schematic of the circuit diagram used for the memristor to process neural signals. A switch (represented by a red bar) is used to control the connection of the memristor to the neural probe or the read circuitry. The switch switches from position **1** (connected to the neural probe to apply the neural spikes to excite the memristor) to position **2** (connected to the read circuitry with a (-30 mV, 0.5 ms) pulse generator) every 20 ms.

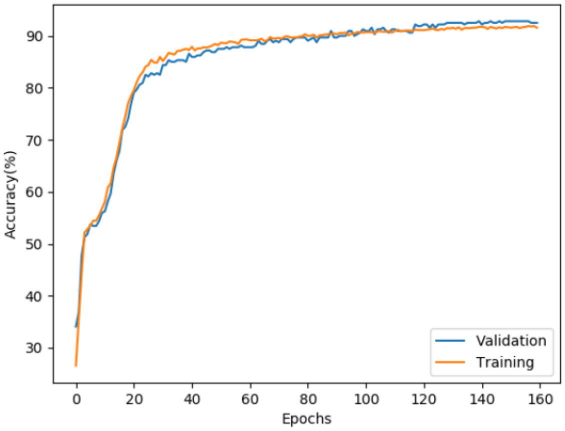

Supplementary Figure 12 Evolution of the classification accuracy with training epochs for the readout layer. A recognition accuracy of ~92.5% was achieved after training.

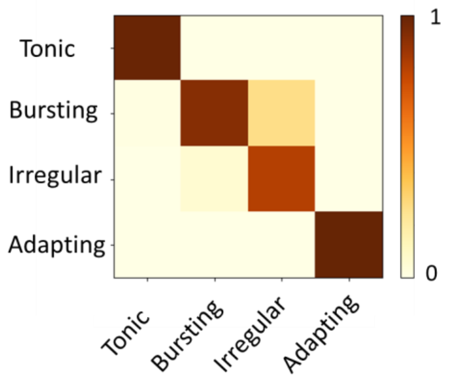

Supplementary Figure 13 False color confusion map showing the pattern recognition result against the ground truth result for each firing pattern.

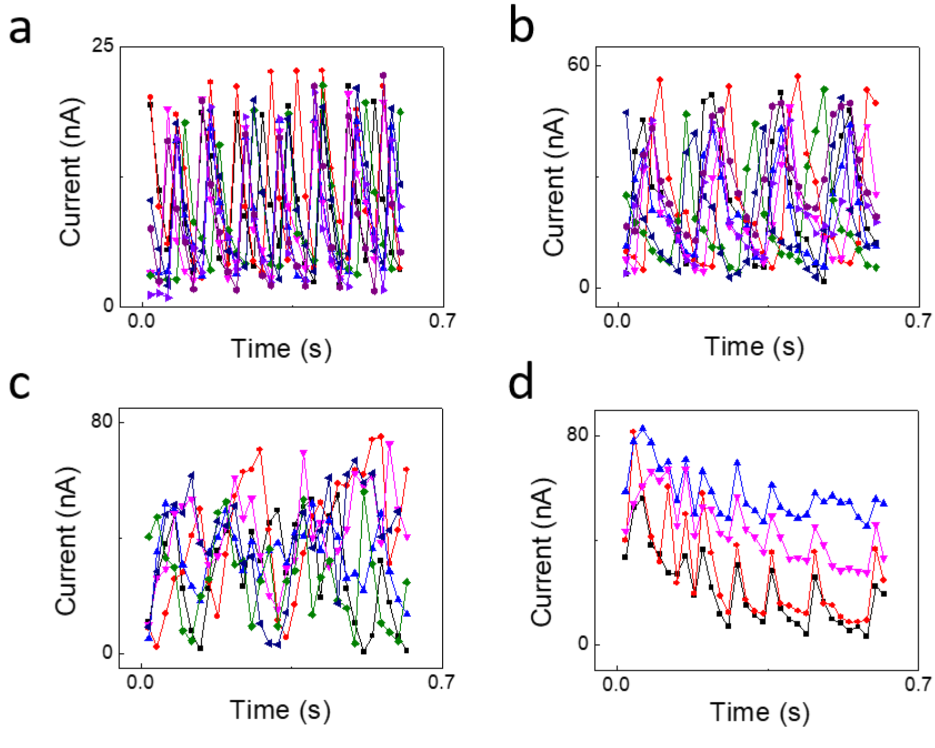

Supplementary Figure 14 Experimentally measured device read currents when being stimulated by spike trains having "Tonic" (**a**), "Bursting" (**b**), "Irregular" (**c**) and "Adapting" (**d**) firing patterns.

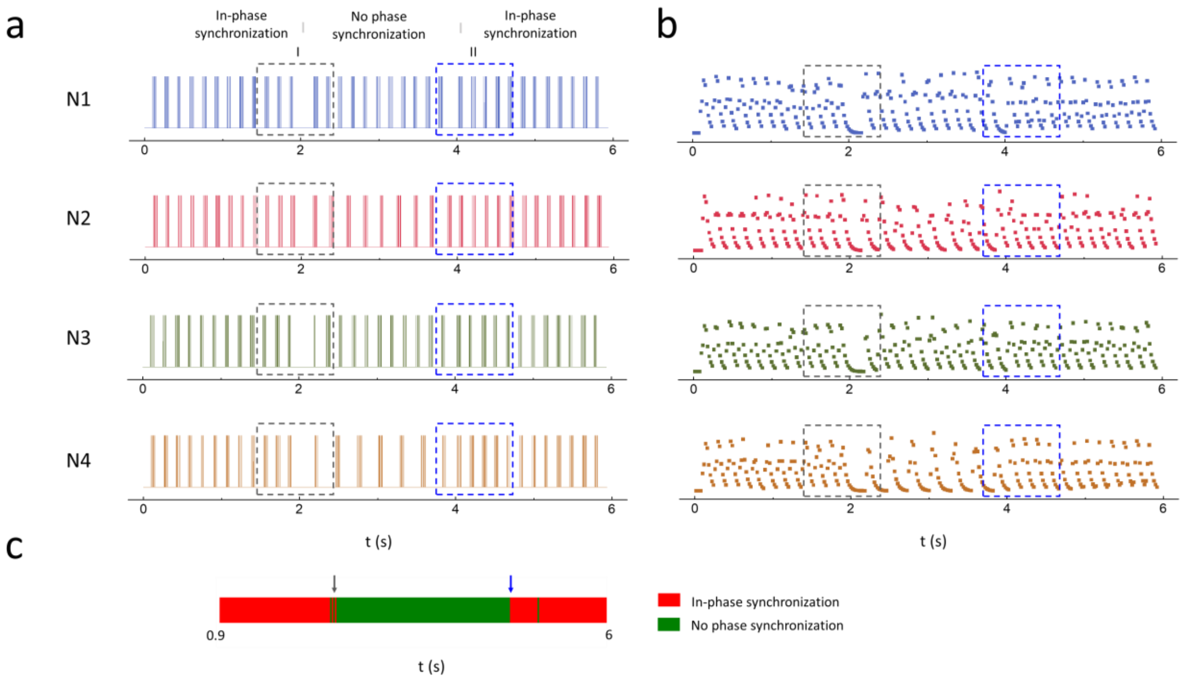

Supplementary Figure 15 Real-time analysis of neural synchronization. **a,** Spike trains showing synchronization states evolving from in-phase synchronization, no phase synchronization to in-phase synchronization among four neurons (N1 - N4). The gray bars mark the transition between different synchronization states. **b**, The corresponding simulated device responses obtained from four memristors. **c,** Classification results from the RC system. The arrows indicate the moments when the synchronization state transitions began to be successfully detected.

Parameter	Value	Parameter	Value
α	1e-8	λ	1e-3
β	0.5	η	56
γ	1e-5	τ	0.04
δ	4		

Supplementary Table 1. Parameters used in simulation for the Ag/$CsPbI_3$/Ag memristor device.

Supplementary Note 1

1) First, we show that the memristor device presents required internal dynamics. Supplementary Figure 6 shows the response of a $Ag/CsPbI_3/Ag$ memristor device to a pulse stream composed of identical electrical pulses (100 mV, 2 ms) with different intervals (from 2 to 80 ms). The device can be excited by each electrical pulse to a new conductance state, while the final device conductance depends strongly on the temporal patterns of the input pulse stream. When pulses with short intervals were applied, the device conductance continued to increase (red arrows in Supplementary Figure 6b), while the excited device conductance will decay spontaneously if no stimulation was applied within a long time period (black arrows in Supplementary Figure 6b). As explained in the main text and in Supplementary Reference 3, these behaviors are due to the inherent short-term memory effect of the device.

2) Nonlinearity is another important feature of a reservoir, which enables the mapping of inputs that are not linearly separable to the reservoir space that can be linearly separated. We note that the memristor is an inherent nonlinear system, as reflected in the nonlinear equations that govern the device conduction (Eq. (1)) and the internal state-variable (*w*) evolution, both as a function of the amplitude of the stimulation and time (Eq. (2)).

We performed additional experiments to verify the nonlinearity of the memristor device. Supplementary Figure 7 shows the conductance of a $Ag/CsPbI_3/Ag$ memristor as a function of the number of simulation pulses, with identical pulse shapes and pulse intervals. The conductance increases quickly with stimulation in the beginning, and the rate of conductance change gradually reduces even though the same stimulation pulses and intervals are maintained. This nonlinear response with stimulation is a direct result of the competitive behavior of V_I drift and diffusion shown in Eq. (2).

The memristor device also exhibits pronounced nonlinear response to the amplitude of the applied electrical stimulation. Supplementary Figure 8 shows the conductance of a $Ag/CsPbI_3/Ag$ memristor device as a function of the amplitude of the applied electrical pulse (80-160 mV, 2 ms), starting from identical initial conditions. This nonlinear response of the device conductance to stimulation amplitude is mainly a function of the nonlinear (*i.e.* exponential) dependence of the internal state-variable (*w*) on the applied voltage in Eq. (2).

In this study, we mostly used pulses with identical amplitudes, so the nonlinear dependence on the stimulation amplitude is not utilized, while the nonlinear evolution of the internal state variable (*w*) over time plays a main role in building the reservoir.

3) Due to the intrinsic short-term dynamics of the memristor discussed in property 1), the memristor device also natively exhibit the fading memory effect. Fig. 2f in the main text shows the device conductance evolution after being excited by a stimulation pulse. After the initial increase due to the stimulation, the device conductance (measured by the read current) gradually decreases over time. This effect is due to the spontaneous V_I diffusion, as shown in Eq. (2). As noted in the discussions of the dynamic behavior, the fading memory effect is due to the native, internal ionic dynamics of the device, so that this effect can be observed in a single device, without having to use recurrent connects in the network.

4) A good reservoir also exhibits separability, *i.e.* the ability to differentiate inputs with different temporal features using distinct reservoir responses.

We evaluated the separability of our memristor by testing it using pulse streams with different temporal features. In one such test, the pulse stream consists of 3 electrical pulses (100 mV or 0 mV, with pulse width 2 ms, and pulse interval 20 ms). Inputs with different sequences, *i.e.* 001, 010, 100, 011, 101, 110 and 111, are applied, where "1" ("0") correspond to a pulse with an amplitude of 100 mV (0 mV), respectively. Supplementary Figure 9 shows the evolution of memristor conductance during these different pulse streams. After the application of the different pulse streams, distinct final memristor conductance values, representing the memristor states, can be clearly obtained (dashed box), demonstrating good separability of the memristor based reservoir.

5) Echo state property of a reservoir refers to the effect that the past inputs affect the present reservoir state, and the influence asymptotically fades out over time.

Supplementary Figure 10 shows a test on the echo state property of the Ag/$CsPbI_3$/Ag memristor device. It plots the evolution of the memristor conductance after being stimulated by two electrical pulse sequences, separated by different intervals. Each pulse sequence consists of a pair of identical electrical pulses (100 mV, 2 ms, with 10 ms interval), and the intervals between the two sequences increases from 30 to 90 ms, as shown in Supplementary Figure 10a-c. As expected, during each sequence, the second pulse (which follows closely the first pulse) always excites the device to a higher conductance state. When the interval between the two sequences after the second sequence is significantly enhanced, caused by the ion accumulation effect discussed earlier. However, due to the inherent short memory effect, the effect of the first pulse sequence gradually decreases as the interval increases, and the effect of the first sequence can be neglected when the interval is increased to 90 ms, as shown in Supplementary Figure 10c. Similar effect can also be observed in Fig. 2g-i in the main text. These results are consistent with the echo state property of the reservoir.

These experimental results discussed above verify that the Ag/$CsPbI_3$/Ag memristors offer the properties of a reservoir, and allow us to build RC systems using such devices.

Supplementary Note 2

The results in Fig. 2g-i in the main text can be understood based on the dynamics of V_Is in the $CsPbI_3$ based memristor. Specifically, for pulse stimulation occurred in the recent past, 1) if the pulses were applied at a high rate, the V_Is generated by each pulse can be accumulated and result in a quick increase of the device conductance; 2) if the pulses were applied at a low rate, the large pulse interval would provide sufficient time for V_I diffusion, which suppressed V_I accumulation and continued increase of the device conductance. If the pulse stimulation occurred in the far past, the V_Is would have fully diffused and cannot affect the present memristive state.

Supplementary Note 3

The memristive effect in $Ag/CsPbI_3/Ag$ devices is attributed to the V_I movement, due to V_I drift by the applied electric field and spontaneous V_I diffusion. As discussed in the main text, the electric field can drive the migration of iodine ions in the $CsPbI_3$ film to the Ag electrode, resulting in the accumulation of large amounts of V_Is at local regions in the $CsPbI_3$ film. These V_Is serve as n-type dopants to form conduction channels that increase the device conductance. Following the physical model of dynamic memristor devices based on V_O migration[4,5], the conduction of the device can be described as

$$\mathrm{I} = w\gamma \sinh(\delta V) + (1-w)\alpha[1-\exp(-\beta V)] \quad (1)$$

which includes the contributions from two aspects: the first term represents the conduction of the V_I-rich region which shows a tunneling behavior and the second term represents the conduction of the V_I-poor region which shows the Schottky emission behaviors[5]. The overall device conductance is determined by the state variable *w*, which corresponds to the area ratio of the V_I-rich region in the device[5].

After the applied electric field is removed, the V_Is will spontaneously diffuse away, resulting in the decay of the conduction channel area and the device conductance. The dynamic equation of *w* can be described as

$$\frac{dw}{dt} = \lambda \sinh(\eta V) - \frac{w}{\tau} \quad (2)$$

which includes two competing factors, *i.e.* the drift of V_Is controlled by the applied electric field (the first term) and the spontaneous diffusion of V_Is (the second term)[5]. Indeed, this model has been successfully used to predict the memristive behaviors in oxide based memristors such as $W/WO_x/Pd$[3,5]. Note that α, β, γ, δ, λ , η and τ in Eq (1) and (2) are parameters that can be affected by the material property.

Simulation of the device responses to the pulse inputs are performed by feeding the pulse parameters including the voltage amplitude, duration and pulse interval to Eq. (1) and (2), followed by self-consistently solving Eq. (1) and (2) to obtain the corresponding read currents of the memristor.

The parameters used for the simulation are shown in Supplementary Table 1.

Supplementary Note 4

We note that in the reservoir computing system, the memristor based reservoir can continuously sense and process the neural signals in real-time, then produces a discrete set of reservoir states such that a relatively small readout neural network is sufficient for the neural data analysis. For example, in our implementation the reservoir states are measured every 20, 40 and 75 ms, such recordings are applied to the 30x4, 15x4 and 8x4 readout network, respectively, for neural signal analysis (Fig. 3f in the main text). In contrast, without the reservoir a high sampling frequency, *e.g.* 10 kHz, is required for neural data recording to ensure the capture of the short but infrequent spikes that cannot be predicted. This process will generate a large amount of data that will be 2-3 orders of magnitude higher than that used in the reservoir computing system, and will in turn require a much larger readout network to process.

One common approach to avoid recording large amounts of data is to use analog or digital circuits that produce an integrated signal of the input data over a pre-determined time period. This approach (termed integrated approach) is equivalent to producing the average signal of the input data over the pre-determined time period, which is a common down sampling technique to reduce the input size. Comparison against the integrated approach shows the reservoir computing system systematically exhibits an advantage of 3-4% higher accuracy using the same-sized readout layer. The improved performance of the reservoir system can be attributed to the separability property of the reservoir shown in Supplementary Figure 9, while the integrated approach cannot distinguish the temporal sequence of spikes during the integration period.

Supplementary References

1. Tappertzhofen, S., Mündelein, H. , Valov, I. & Waser, R. Nanoionic transport and electrochemical reactions in resistively switching silicon dioxide. *Nanoscale* **4,** 3040-3043 (2012).
2. Ge, S., Huang, Y., Chen, X., Zhang, X., Xiang, Z., Zhang, R., Li, W. & Cui, Y. Silver Iodide Induced Resistive Switching in $CsPbI_3$ Perovskite‐Based Memory Device. *Adv. Mater. Interfaces* **6,** 1802071 (2019).
3. Du, C., Cai, F., Zidan, M. A., Ma, W., Lee, S. H. & Lu, D. W. Reservoir computing using dynamic memristors for temporal information processing. *Nat. Commun.* **8**, 2204 (2017).
4. Chang, T., Jo, S.-H., Kim, K.-H., Sheridan, P., Gaba, S. & Lu, W. Synaptic behaviors and modeling of a metal oxide memristive device. *Appl. Phys. A* **102,** 857-863 (2011).
5. Du, C., Ma, W., Chang, T., Sheridan, P. & Lu, D. W. Biorealistic Implementation of Synaptic Functions with Oxide Memristors through Internal Ionic Dynamics. *Adv. Funct. Mater.* **25**, 4290-4299 (2015).
