# Supplementary material for: Memristor networks for real-time neural activity analysis
Source: Nat Commun. 2020 May 15;11:2439. doi: 10.1038/s41467-020-16261-1 (PMC7228921; doi:10.1038/s41467-020-16261-1)
Supplement: Supplementary file 2 — Description of Additional Supplementary Files [file 41467_2020_16261_MOESM2_ESM.pdf]

Description of Additional Supplementary Files

File name: Supplementary movie 1
Description: Real-time neural firing pattern recognition
