# Supplementary figures and images for: Memristor networks for real-time neural activity analysis
Source: Nat Commun. 2020 May 15;11:2439. doi: 10.1038/s41467-020-16261-1 (PMC7228921; doi:10.1038/s41467-020-16261-1)

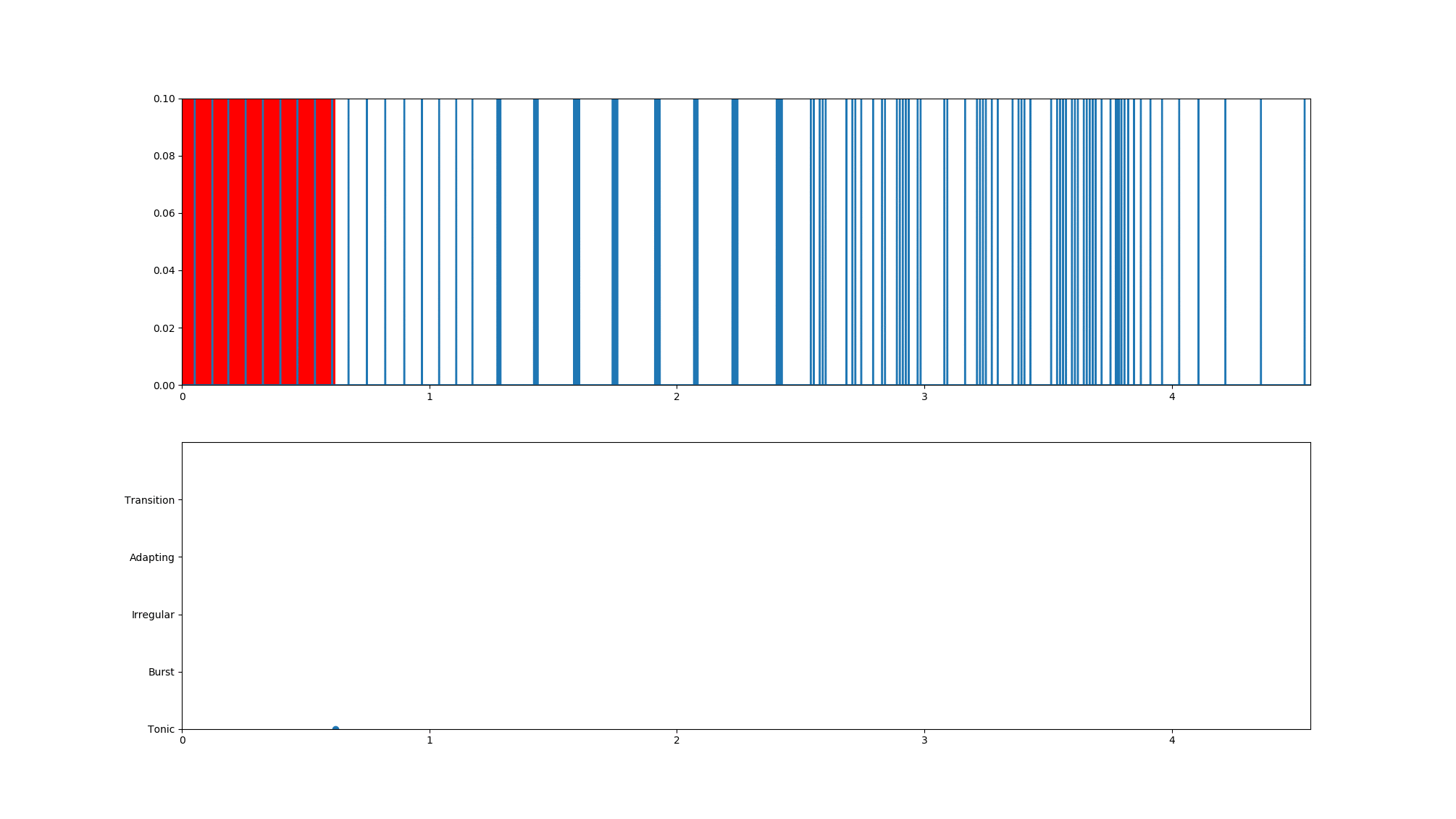

Supplement: Supplementary file 3 — Supplementary Movie 1 [file 41467_2020_16261_MOESM3_ESM.gif]
